# “More players can reach national and international levels”: coaches perceptions of “birthday-banding” in youth squash and its potential for minimising relative age effects

**DOI:** 10.3389/fspor.2025.1618333

**Published:** 2025-07-25

**Authors:** Adam L. Kelly, Achuthan Shanmugaratnam, Kathryn Johnston, Joseph Baker, Matthew Ferguson, Mark Jeffreys, Josh Taylor, Alexander B. T. McAuley

**Affiliations:** ^1^ Research for Athlete and Youth Sport Development (RAYSD) Lab, Research Centre for Life and Sport Sciences (CLaSS), College of Life Sciences, Birmingham City University, Birmingham, United Kingdom; ^2^ Tanenbaum Institute for Science in Sport, University of Toronto, Toronto, ON, Canada; ^3^ University of Birmingham, Birmingham, United Kingdom; ^4^ England Squash, National Squash Centre, Manchester, United Kingdom

**Keywords:** athlete identification, athlete selection, athlete development, talent identification, talent selection, talent development

## Abstract

Relative age effects (RAEs) are common across many youth sports that use age group structures to band athletes. This creates a significant overrepresentation of those who are born near the start of the selection cut-off date across talent pathways compared to those born towards the end. In an attempt to identify, select, and develop the most talented squash players based upon their long-term potential, England Squash designed and implemented the “birthday-banding” approach (i.e., athletes compete with and against those of the same age and move up to their next birthdate group on their birthday), which has indicated promising results for moderating RAEs across their player pathway. However, little work has focused on the perceptions of interest-holders on this approach. For this reason, the purpose of this study was to use semi-structured interviews with fifteen England Squash Talent Pathway coaches, to better understand the mechanisms of the birthday-banding approach as well as its potential benefits and limitations. Using thematic analysis, three higher-order themes were found that comprised of six lower-order themes: (a) considering organisational structures (e.g., understanding selection processes, and reflecting on competition structures and performance outcomes), (b) building appropriate settings (e.g., promoting flexibility and fluidity in groups, and creating an environment that fosters long-term development), and (c) facilitating individual athlete development (e.g., encouraging holistic development and progression, and evaluating physical and skill development). Overall, coaches spoke highly of the implementation of birthday-banding, noting the value in creating fairness for athletes who might have been removed due to their birthday and maturation levels. Coaches also reported appreciating seeing athletes having varying competition within and across a year, as sometimes athletes would be relatively older and younger than their peers within the same 12 months. Some considerations and concerns were also raised about implementing a birthday-banding approach, which have been highlighted to inform continued improvements for both athletes and coaches in the system.

## Introduction

The purpose of talent development systems is to provide young athletes with a suitable learning environment to accelerate or realise their potential (1). However, when identifying (i.e., recognising individuals with the potential at an earlier age to become high performers in the future) and selecting (i.e., ongoing process of identifying individuals at various stages of development who demonstrate prerequisite levels of performance) athletes into these systems (2), there can be a range of personal- and system-level challenges that can result in biases and inaccurate decisions (3). One such bias that can influence these key processes is reflected in the well-known relative age effects (RAEs). This phenomenon refers to the overrepresentation of relatively older athletes (i.e., those born near the start of the cut-off date, such as January 1st) and underrepresentation of relatively younger athletes (i.e., those born near the end of the cut-off date, such as December 31st) in team rosters when individuals compete in fixed annual age groups in youth sport (e.g., U10, U11, U12, etc.), (4, 5). Such effects are evident from early in the pathway, as recreational and developmental leagues and programs often use age cut-offs (6). This has a pronounced knock-on effect on those who are subsequently recruited into talent development systems at young ages where RAEs become even more significant (7).

Relative age effects are almost ubiquitous throughout talent development systems [see (8, 9) for reviews]. Indeed, research has showed they are prevalent across many team sports [e.g., handball; (10)] and individual sports [e.g., swimming; (11)], as well as in physical education [e.g., academic achievement and school sport representation; (12)] and in comparatively less physically active domains [e.g., cognitively demanding tasks such as chess; (13)]. As an example, a systematic review on RAEs in female sport (9) showed that, across 57 studies (*n* = 646,383), athletes born in birth quarter one (BQ1; i.e., those born in the first 3 months of the annual selection year) were significantly overrepresented (26%) compared to those born in birth quarter four (BQ4; i.e., those born in the last 3 months of the annual selection year) who were significantly underrepresented (22.6%).

Although RAEs have been shown across a range of ages, competition levels, countries, and playing positions in both female and male sports, the mechanisms driving these effects remain mostly hypothetical (14–16). The current consensus is that they occur due to a combination of task (e.g., sport), performer (e.g., playing position), and environmental (e.g., country) constraints (17). These influences are co-dependent on a single cut-off date, which can differ depending on the sport, country, and organisational policies (12, 18). For instance, volleyball in The United States has 17 different cut-off dates alone depending on state regulations; however, RAEs shift according to these timepoints, which has been shown in other sports (18–20, 60). This helps to highlight how a single cut-off date can significantly impact an athlete's identification and selection into a talent development system (16), shedding light on how the current chronological age group system has questionable effectiveness, efficiencies, and ethics in developing talented young athletes.

The same trends can be seen in youth racket sports, with research showing how RAEs are generally consistent across different countries and genders in tennis [e.g., (21–24)], table-tennis [e.g., (25–27)], and badminton [e.g., (6, 28, 29)]. In tennis, for example, relatively older players within an age group are more likely to be selected for development programs (e.g., national team training camps), receiving more training and competition opportunities, which contributes to their continued development and success (21). Similarly, in table tennis, Faber et al. (26, 27) reported consistent relative age patterns across European youth competitions, where earlier born players were more likely to follow higher performance trajectories, whereas fewer relatively younger players reached these elevated levels. Likewise, Bilgiç and Devrilmez (30) showed comparable results in badminton, where RAEs favoured relatively older athletes across both singles and doubles categories during three consecutive European Badminton Championships, with players born in BQ1 and BQ2 over five and four times more likely to reach the podium than those born in BQ4, respectively. Collectively, this research emphasises the inefficiencies of current talent development systems, with age group structures often favouring those who are relatively older and disadvantaging those who are relatively younger.

Although findings are consistent at the youth levels, they appear more variable at senior levels. For instance, Zháněl et al. (24) and Bilgiç and Güvenç (25) showed significant RAEs at senior levels in tennis and badminton, respectively. As an example, Zháněl et al. (24) examined senior female tennis players ranked in the world top 100 from 2007 to 2016, showing RAEs for those born earlier in the year (e.g., BQ1 and BQ2), with further significant RAEs present among the world top 10 players. Interestingly, significant RAEs were also observed in older age subgroups (e.g., aged 19–36 years), suggesting that the impact of RAEs may intensify and persist into later stages of an athlete's career (24). In comparison, however, Ulbricht et al. (23) and Romann et al. (6) showed no RAEs at senior levels in tennis and table tennis, respectively. Ulbricht et al. (23), for instance, found little evidence of RAEs in senior ranked (56% born in the first half of the year) and senior recreational (49% born in the first half of the year) German tennis players.

These inconsistent findings are likely due to a combination of factors, including gender and sport popularity. For example, gender has been shown to moderate RAEs, with males generally experiencing stronger effects than females, which is possibly a result of higher competition levels and greater selection pressure in male sports (6, 8). Relatedly, Romann et al. (6) conducted a nationwide analysis of youth athletes across 68 male and 63 female sports in Switzerland, revealing RAEs were more pronounced in male athletes, especially in sports with higher participation and competitive depth. Conversely, RAEs in female sports were small and largely consistent across selection levels. Moreover, sport popularity has been shown to influence the extent to which RAEs are prevalent, whereby the more popular the sport is and the more competition there is for the limited competitive places at the highest levels of engagement in the sport, the higher RAEs become, and vice versa [e.g., (6, 8, 31)]. For instance, Romann et al. (6) showed a “residual bias” (i.e., the persistent overrepresentation of relatively older athletes in adult sport, where the expected or obvious effects of relative age should have faded) in Olympic sports compared to non-Olympic sports. This was attributed to the greater popularity, visibility, and funding of Olympics sports, thereby attracting larger athlete pools and creating higher selection pressures ultimately intensifying RAEs more at youth levels compared to non-Olympic sports, which has a subsequent knock-on effect in the longer-term (i.e., at adulthood).

There is one racket sport, however, that has shown no RAEs to date, across both youth and senior cohorts as well as for male nor female talent pathways—squash (32). Squash is an important sport to monitor from a relative age standpoint, since it is played globally and is particularly popular in England, where two players are, at the time of writing (33), ranked in the top 20 internationally for both women and men. Due to its popularity and international success, a greater emphasis has been placed on structured development for athletes at an early age. This has led to the creation of the England Squash Talent Pathway, which prioritises the identification, selection, and development of the best players in the country to help them succeed on the international stage. Interestingly, when examining RAEs across the five steps of their England Squash Talent Pathway, no effects were found across their mixed-gender pathway (BQ1 = 21% vs. BQ4 = 24.7%) or across genders (total female BQ1 = 21.4% vs. BQ4 = 23.8%; total male BQ1 = 20.3% vs. BQ4 = 25.2%). Its growing interest from a participation and spectator standpoint, combined with the recent news of its debut in the 2026 Olympics, make for an important case study to examine.

**Figure 1:**
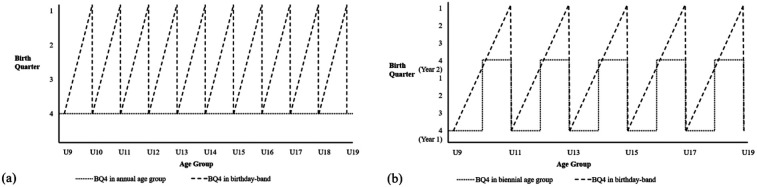
The U9 to U19 birthday-banding developmental trajectories compared with **(a)** annual age grouping, and **(b)** and biennial age grouping. Note, the example comparisons use a BQ4 athlete for the annual age group and biennial age group [adapted from (34)].

The encouraging absence of RAEs across the England Squash Talent Pathway is likely attributed to their novel grouping approach, referred to as “birthday-banding” (34). Birthday-banding involves athletes competing with and against those of the same age (i.e., all the 13-year-olds compete together, all the 14-year-olds compete together, etc.) and move up to their next birthdate group on their birthday, rather than competing in fixed chronological age groups (i.e., U13, U14, U15, etc.). The aim of birthday-banding is to remove specific selection time-points, maintain recruitment on a continual basis, and ensure there is an equal opportunity for all players to be selected during the entire selection year (32). The birthday-banding strategy also affords more diverse experiences, by allowing individuals to be both relatively older and relatively younger throughout their development. As a practical example, if a young athlete was born in August in England and competed in fixed chronological age group structures, they would be a BQ4 throughout their entire youth development. In contrast, if the same athlete was to compete in birthday-banding structures, they would start as a BQ4 in August (on their birthday), and then gradually progress towards being the oldest (i.e., BQ1) until the end of their 12-month development (on their next birthday) where they would again become the youngest (see Figure 1 for a visual representation) (34). In addition to its potential positive impact on mitigating RAEs, birthday-banding may provide additional benefits such as drawing unique benefits from mixed age play (35), enabling different types of social comparison environments (36), and moderating other identification and selection biases such as relative growth effects (37, 38).

Importantly, however, many of these suggestions remain hypothetical, as perceptions of birthday-banding are yet to be explored from various interest-holders. For example, Bilgiç and Güvenç (25) reported no significant RAEs among senior squash players in Turkey, attributing this to the relatively low popularity of squash in the country and the smaller athlete pool compared to other racket sports. The limited competition for places likely reduced selection pressures, thereby minimising the impact of RAEs. Moreover, qualitative methodologies in relative age research is generally lacking (39, 40). To fill this void, the purpose of this study was to better understand the perceived operational mechanisms of “birthday-banding” as well as the perceived player development outcomes associated with the birthday-banding approach. Using semi-structured interviews, coaches working throughout the England Squash Talent Pathway were invited to share their experiences and ideas with respect to the birthday-banding practices in squash. It was hypothesised that coaches would view birthday-banding in a positive manner with respect to its effect on the player development environment, emphasising its ability to create more diverse settings that focus on each individual's needs, and, in turn, highlight its ability to moderate RAEs during identification, selection, and development processes.

## Methodology

### Research paradigm

The current study will be guided through a constructivism research paradigm with a relativist ontological position and subjectivist and transactional epistemological position. Within a relativist ontological position, it is assumed that there is no single external reality independent of the individuals, rather reality is seen as multiple individual mental constructions about the world, shaped through lived experiences (41). A subjectivists and transactional epistemological position assume that knowledge is co-created through interactions between the researcher and the participant, whereby the researchers bring their past experiences and interpretations to the study (42). This theoretical perspective was fitting for our research question under examination because it allowed us to explore how birthday-banding is perceived by key interest-holders (i.e., coaches), through their lived experiences and interpretations. Rooted in the subjective realities of coaches, our study seeks to understand how their knowledge and experiences shape their perceptions, while also acknowledging the role of the researchers in guiding discussions, and interpreting and constructing meaning from the data (43).

While guided by these philosophical and theoretical positions and values, the chosen method for knowledge gathering was though qualitative interviews with coaches who have experience using, working within, and applying the birthday-banding in their work with athletes. To do so, semi-structured interviews were utilised as a way to illicit storytelling, experience sharing, and conversations between the coaches and the interviewer. The dynamic nature of semi-structured interviews allowed participants a degree of freedom to discuss experiences most relevant to them with the birthday-banding approach as a coach (44). This technique also allows for greater flexibility and creativity in the interview process compared to an approach like a structured interview, as appropriate times during the interview, the interviewer (MF) was able to probe with related questions, to further unpack insightful details from participants' responses to questions. Using this method aligns with our guiding paradigm that amplifies the many voices and perspectives of a phenomena such as a birthday-banding (and more generally talent selection) in the context of sport.

### Positionality

Bourke (45) describes how researchers often co-develop knowledge with research participants based on their positionality in association with the given questions within a project. In fitting with our philosophical position of constructivism with subjectivists and transactional views, the experiences and knowledge of the interviewer coming into (and during) the knowledge gathering process through interviews was celebrated rather than diminished. In this case, the primary interviewer, did not have any experience within the sport of squash and the practices of birthday-banding.

### Participants

After obtaining ethical approval from the Health, Education, and Life Sciences Faculty Academic Ethics Committee at Birmingham City University (reference code: #3293), the authors used purposeful sampling to recruit 15 England Squash Talent Pathway coaches from United Kingdom, aged 29–49 years. All 15 coaches were emailed invitations and accepted to participate. Amongst the 15 coaches who participated in this study, 14 identified as male, and one identified as female. All coaches had extensive experience in squash coaching with years of experience ranging from 10 to 32 years. Among the participants, nine individuals held the England Squash Level 4 qualification and six participants had achieved the England Squash Level 3 qualification, demonstrating advanced expertise in coaching in squash at high performance levels. The coaches worked with athletes of various ages, ranging from as young as 3 years old to adults in their 70s, and coach players at different competitive levels, including beginners to professional and international levels. The average weekly squash coaching hours ranged from 15 to 40 h per week. Importantly for this study, all coaches had experience either being directly involved in, or working within the England Squash Talent Pathway that used the birthday-banding approach.

**Figure 2:**
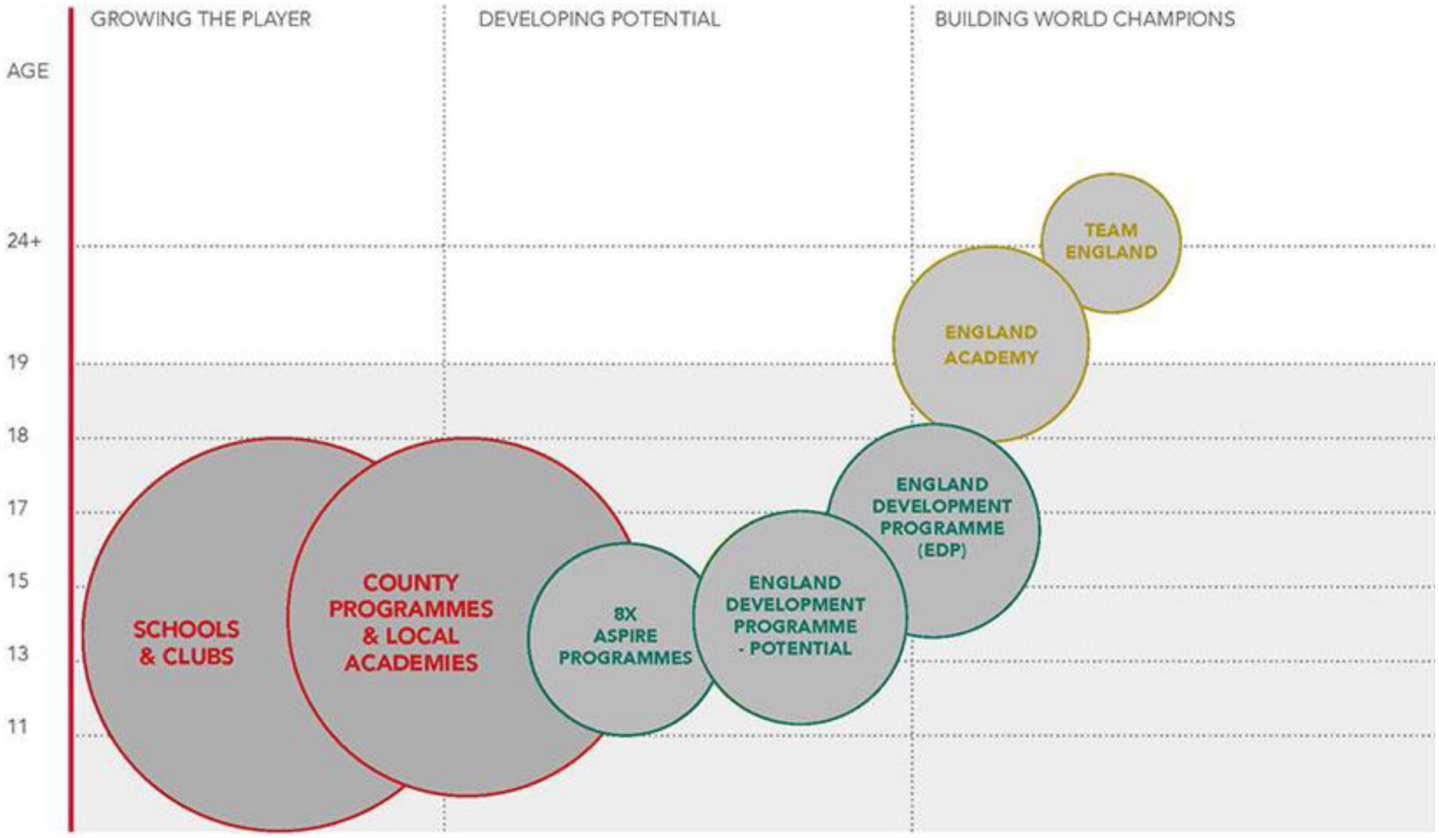
The England squash talent pathway [adapted from (32)].

Figure 2 provides an overview of the England Squash Talent Pathway. ASPIRE provides the first stepping-stone onto the England Squash Talent Pathway for those who are mostly selected from aged 11 to 16 years, offering the most promising young players an environment to develop across eight English regions. This feeds into the Potential group, focused on providing the first national level squad for the younger and developing talent in England. This develops and leads individuals into the Development group, which supports individuals towards a world class level for the England Academy and England Senior Team (46). Training time varies across the five selection levels: (a) ASPIRE = 3–6 h/week; (b) Potential = 5–10 h/week; (c) Development = 7–14 h/week; (d) Academy = 15–20 h/week; and, (e) Senior Team = 15–20 h/week.

### Data collection

An interview guide was developed with inspiration from the work of Goldman et al. (47), whose interview examined athletes' perceptions of “playing-up” (i.e., athletes competing in an older age group) in youth football. The interviews began with some broader opening questions to set an inclusive and friendly environment (i.e., “Tell me about your role in the organisation and how you started coaching squash”). They then progressed to an introduction into the relevance of birthday-banding within the England Squash Talent Pathway specifically (i.e., “How is the concept of birthday-banding relevant to your organisation?”). An introduction to the topic was then followed by the general discussion of birthday-banding, which entailed various aspects including its implementation (i.e., “How was birthday-banding put into practice in your organisation?”), and evaluation (i.e., “What was the impact of using birthday-banding?”). The interviews were then concluded with a closing statement, thanking the coach for their participation in the interview, as well as allowing the opportunity for the participant to ask any questions or add any relevant information which may have not been covered through the interview questions (i.e., “Is there anything that you would like to mention that we haven't covered already?”). Using this approach, the third author facilitated participants' insights of experiences and perceptions on the birthday-banding approach in their own coaching environments. Before commencing main interviews, two pilot interviews were conducted with two squash coaches from the England Squash Talent Pathway. These pilot interviews were not included in the data analysis as they served to refine the interview approach, ensuring clarity and relevance of the questions in order to yield rich data for the analysis.

As part of the data collection process, the interviewer video and audio-recorded 15 interviews with 15 unique squash coaches as part of the data collection process. All interviews took place online via Microsoft Teams (Microsoft Corp., Redmond, WA, The United States), with only the interviewer and the respective participants present. On average, the interview length was 39.87 (SD = 14.26) minutes. Upon completion of all interviews, the interviewer transcribed the interviews using Microsoft Teams Transcriber (Microsoft Corp., Redmond, WA, The United States). Confidentiality of coaches who participated in the interview was maintained by assigning a random participant number to each coach to de-identify the transcribed audio data collected.

In addition to the interview transcripts, the interviewer kept a reflexive journal. Throughout each interview, observation of the coaches' and notes were made (e.g., tone, body language, ease or difficulty when participants responded to questions). Upon the completion of each interview, key messages, and overall thoughts of the interview were recorded by the third author through his notes which were ultimately synthesised to form the reflexive journal. This process is particularly important for qualitative research to maintain transparency and sincerity during the data analysis process and to document and reflect on personal assumptions (48).

### Data analysis

Once participant interviews were transcribed, the second author conducted an inductive thematic analysis of the transcripts to identify and categorise emerging themes from participants' interviews (49). Informed by Braun et al.'s (49) six-step process, the second author conducted the thematic analysis on Microsoft Word (Microsoft Corp., Redmond, WA, The United States). To begin (i.e., step 1), the second author familiarised themself with the data by reading over the transcripts. Next (i.e., step 2), using the comment function on Microsoft Word, the second author “tagged” pieces of the text that were relevant to the research purpose with one or more code. Then (i.e., step 3), to begin constructing lower-order themes, they examined codes and associated data to cluster them into bigger codes or provisional themes to capture meaningful patterns in the transcripts. Following this (i.e., step 4), they reviewed the provisional lower-order themes. This included ensuring all codes were representative of the lower-order theme and the lower-order themes were relevant to the research question. To capture an idea of an underpinning group of lower-order themes, higher-order overarching themes were also developed. Subsequently (i.e., step 5), they defined and named these themes. Finally (i.e., step 6), they compiled and revised all analytic writing to integrate it into a final report.

### Methodological rigor

To ensure effective and ethical qualitative research practices, the authors employed a study design in accordance with criteria for excellent qualitative research [see (48)]. Tracy (48) proposed a model entailing eight key indicators of quality in qualitative research. Based on this work, the following criteria contributed to rigor in this study: worthy topic, significant contribution, sincerity, meaningful coherence, and credibility. Given the scarcity of literature surrounding the birthday-banding approach in youth sports, the present study covered a worthy topic. To the authors' current knowledge, the coaches' perception of birthday-banding approach in youth sports (within any sport) has yet to be examined. Indeed, the coach is a pivotal interest-holder in the birthday-banding approach, as they are often the ones responsible for identifying athletes, making (de)selection decisions, and subsequently working with those athletes in a training and competition environment Understanding the perceptions of the coaches may help to inform theory, shape coach education, challenge and inform selection policies, and support coaching practices within (and perhaps beyond) the sport of squash.

The authors recognise they are coming into this work with preconceived notions and experiences that will shape the way the study is conducted, and have try to stay transparent and sincere throughout the work. Throughout the data collection and analysis process the interviewer utilised the reflexive journal to record any personal assumptions. To accomplish meaningful coherence (i.e., ensuring the study aligns its aims, methods, and findings), the authors collaborated to ensure the construction of an effective interview guide that aligned with the purpose of the study. Lastly, to accomplish credibility, interviews were held with as many coaches as possible given the purposeful sampling. Having 15 coaches who were experienced and knowledgeable on the topic, working with some of the most elite athletes for their age in the country, helps to illuminate an important perspective on how birthday-banding is used. These coaches were directly involved with the birthday-banding approach in England youth squash, leading to rich data informed by coaches with lived experiences. In addition, a degree of alignment in the labelling and coding was achieved (i.e., triangulation), through multiple iterative consensus meetings among the authors. These meetings focused on reviewing and refining the coding and thematic structure from the data analysis based upon the contents from the interview transcripts.

## Results

**Figure 3:**
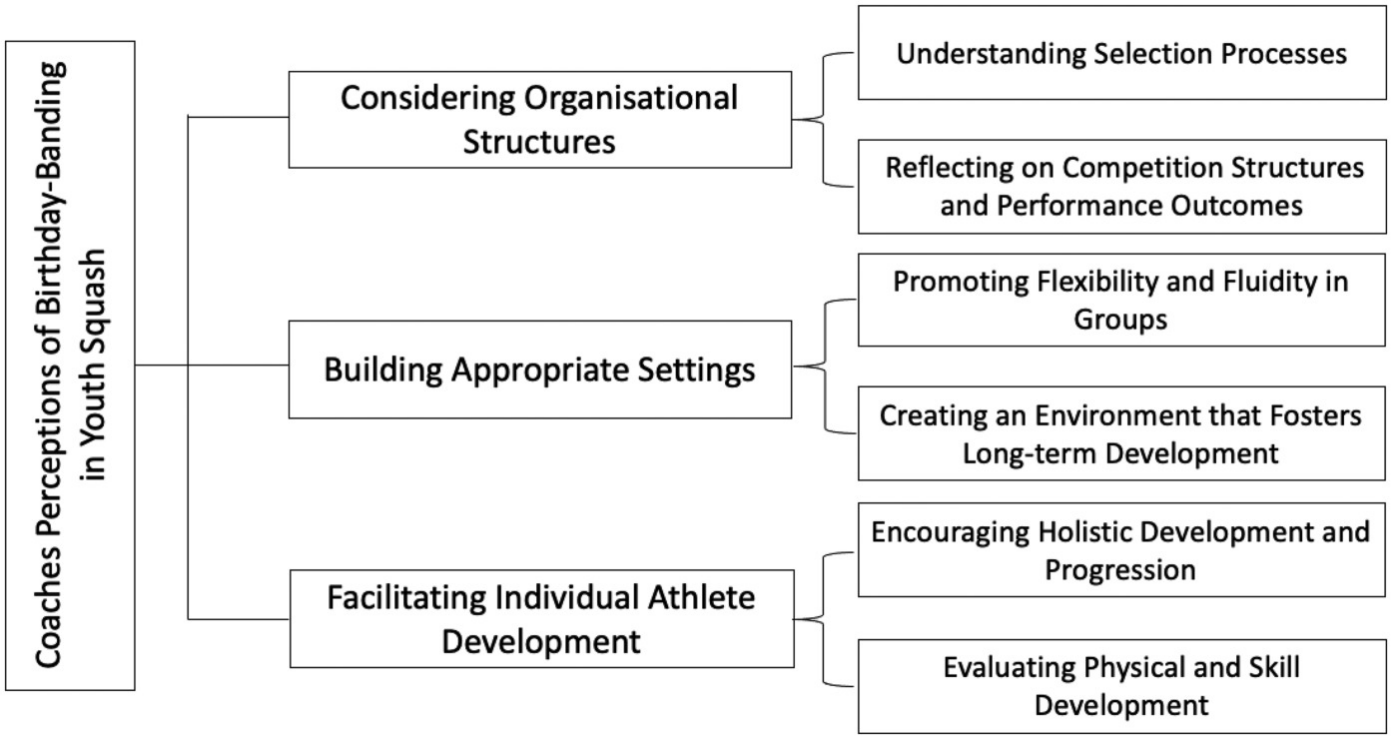
The three higher-order themes comprised of six lower-order themes based upon coaches’ perception of birthday-banding in youth squash.

Participant discussions centred around the operational mechanisms of birthday-banding and its associated player development outcomes, highlighting three higher-order themes comprised of six lower-order themes: (a) considering organisational structures (e.g., understanding selection processes, and reflecting on competition structures and performance outcomes), (b) building appropriate settings (e.g., promoting flexibility and fluidity in groups, and creating an environment that fosters long-term development), and (c) facilitating individual athlete development (e.g., encouraging holistic development and progression, and evaluating physical and skill development) (see Figure 3). These higher-order themes reflect organisational, environmental, and individual outcomes inherent with youth sport development, respectively.

### Considering organisational structures

Participants frequently highlighted the importance of considering organisational structures as a critical factor with the implementation of the birthday-banding approach. These considerations reflected the selection processes as well as competition structures and performance outcomes.

#### Understanding selection processes

Participants believed the birthday-banding approach held important implications for the selection processes in England Squash. Participants mainly highlighted the more inclusive and development-focused opportunities for younger players available through selection processes. Several participants spoke positively about how the birthday-banding approach allows interest-holders involved in the selection process to identify and nurture the potential of young players beyond traditional age-grouping constraints. For example, Participant 12 highlighted the flexibility associated with birthday-banding, which allowed younger players who may be overlooked to have the opportunity to be involved in high performing environments:

“I'd say that birthday banding provides a lot of flexibility in squad selection processes, especially for youth players. It opens up opportunities for young players with potential to be involved in these environments, which is a real positive. I've seen many players develop rapidly and gain confidence. Coaches can select players based on attributes that they believe will help long-term development rather than being constrained by a ranking system” (Participant 12).

This example highlights the strong perceptions participants held on the positive association between birthday-banding and the selection process, specifically relating to the opportunities for young players who may be overlooked to be selected into further developmental or high-performance programmes. As Participant 12 noted, birthday-banding allowed for the emphasis on selecting players based on attributes indicative of high potential, rather than focus on the current performance and outcomes, which can be skewed by relative birth advantages such as maturation and growth.

With focus shifting to players' abilities within a birthday band rather than their immediate performance in selection processes, multiple participants believed this approach created greater opportunities for later developers or relatively younger players. Specifically, participants 5 and 8 believed that if relatively younger players demonstrated high potential through key attributes relevant to squash (e.g., effective use of different strokes), they were more likely to be recognised and given a chance to develop within a birthday-banding system rather than a traditional age-grouping system.

Whilst positive advantages in the selection process were mainly highlighted by participants when discussing the birthday-banding approach, some also believed that unique disadvantages existed for players in the selection process existed depending on their specific date of birth. For example, Participant 14 noted that when athletes try and represent England, some interest-holders may tend to favour the selection of players born towards the end of the season (i.e., April to July) due to the timing of international events and players born in this specific time range being at the peak of their performance. Participant 15 added, “It would be a shame for someone to miss out on an invitation to a national squad simply because they were a couple of weeks short of qualifying. I know of players who have just missed cut-offs for events and end up missing out on opportunities because of timing”. This example highlights participants 14 and 15 believed certain players had unique advantages in the selection process, especially if their date of birth closely aligned with timing of key competition events.

Participants 2, 5, 8, 12, 14, and 15 believed if coaches and key interest-holders involved in the selection processes understood and were aware of these advantages, it would help minimise barriers in the selection process for players not born in these optimal time ranges. Participant 15 added “players born closer to competition dates may have more chances to gain ranking points. This could open doors to more squads, but we must be careful about how we assess potential based on timing”. Indeed, participants 14 and 15 agreed that certain advantages may exist in the selection process for players born at an optimal time (e.g., being born closer to competition dates), however, they also noted to minimise the impact of a player's birthdate influencing selection opportunities, coaches and those involved in the selection process need to be aware of such biases and reflect on their selection criteria and process accordingly. Additionally, most participants strongly believed that biases related to a player's birthdate (i.e., RAEs) or relative birth advantages, such as maturation and growth, were more easily recognisable within a birthday-banding approach. This increased awareness enables coaches to make more informed selection decisions, ultimately minimising the negative effects associated with birthdate disparities.

Despite the potential negative impact of birthday-banding on the selection process as highlighted by a few participants, most believed these challenges could be addressed. Participants suggested that if coaches can understand the selection process and established possible barriers (e.g., unfavourable birthdays), they could adopt a more flexible approach. By integrating players based on their potential, recognised through key attributes or characteristics rather than immediate performance, the limitations of the birthday-banding system could be minimised. Overall, the examples and positive perceptions shared by participants about the impact of the birthday-banding approach on selection processes suggest that it is seen as a valuable tool for talent identification.

#### Reflecting on competition structures and performance outcomes

Participants noted that the birthday-banding approach was mainly put into practice through competition structures and performance settings, offering a unique approach for player development as it can provide a longer developmental window within an athlete's relevant birthday-band compared to rigid annual age group classifications. This extended period was perceived by participants to allow players more time to develop, compete, and progress within their age group before moving up. For example, a player born later in the selection year may benefit from competing against similar aged peers for an extended period before transitioning to the next age category, rather than being disadvantaged by strict 1-year age classifications.

A key advantage specifically highlighted by participants 1, 2, 4, 7, 12, and 13 with the implementation of birthday-banding approach in competition structures is the opportunities players have to compete against others at different developmental stages, given the biennial birthday-band groups (i.e., U11, U13, U15, etc.). Although the early time spent in a birthday band may be challenging for some players due to greater age differences coupled with physical and maturational variances, this structure and performance outcomes could foster psychological skills, such resilience and motivation. Participant 1 noted:

“Sometimes at tournaments, you'll see a 13-year-old getting completely outmatched by a 15-year-old, simply due to the physical differences. But because this system is in place from an early age, the kids get used to it. They understand that some years will be tough, but that motivates them to keep pushing for the year after, when they'll be among the older ones in their group”.

This example highlights the belief amongst participants that the birthday-banding approach, mainly perceived to be implemented through competition structures, fosters an environment conducive to growth through appropriate challenges within players' relative biennial birthday-banding structure.

Some participants highlighted that competition structures and their schedules could create unique advantages or disadvantages for a player depending on their birthdate. More specifically, if a players' birthdate closely aligns with key competition events that then allows them to compete in competitions when at their strongest, it would be considered a “good” birthdate. For instance, Participant 4 noted, “The only barrier [to birthday-banding] is the tournament calendar. Some kids might miss out on certain events. If a child has an awkward birthdate, they might not get to perform in some events when they're at their strongest”. Participants 12 and 13 added that athletes are quite aware of the unique advantages and disadvantages associated with their birthdates. As an example, Participant 13 added, “They [athletes] often discuss their birthdays and how they relate to tournament schedules. For example, my birthday is on January 4th, and that's considered a disadvantage because I miss out on the British Open, whereas others born on January 10th get to compete in both the nationals and the British Open. They are aware of how their birthdays affect their opportunities in tournaments”.

This disadvantage, however, is somewhat counterbalanced by the flexibility birthday-banding provides alongside competitions occurring year-round, thereby minimising the effect of an “optimal” birthdate, or RAEs, allowing players to compete at various times during the year. Participant 6 highlighted “competitions are year-round. A player might miss one opportunity but could excel in another. You can't control everything, but you can manage the training environment to ensure that birthday differences don't become disadvantages”. Further, Participant 3 added by suggesting rotating major tournament dates (e.g., British Open Junior) to prevent the same player from consistently benefitting from a fixed cut-off date due to birthdate related advantages. Ultimately, participants agreed that while competition scheduling could be refined, particularly by rotating major tournament cut-off dates, the birthday banding approach helps level the playing field over time. Importantly, participants also emphasised that given the benefits of birthday-banding and the year-round competition structure, performance outcomes should be evaluated across all events rather than focusing solely on major tournaments. This broader perspective helps mitigate birthdate related biases when assessing player development.

Within the birthday-banding approach, high-performing players may be placed in older birthday-banding age categories in competitions, a process commonly referred to as “playing-up”. Participants perceived that the flexibility of the birthday-banding approach, allows exceptionally high-performing players to challenge themselves at higher levels in competition structures, ultimately promoting their continued growth. However, participants 8 and 13 both raised concerns about ensuring a balanced competitive environment within each band. Participant 13 noted, if too many top players in a birthday band are playing-up, the remaining competitors in their original birthday band may lack strong opposition, potentially weakening competition within the group. Participant 8 added, “The system seems to be working pretty well. My only concern would be for children who don't participate enough within their banding level. That's dangerous for the overall structure, and I'm not entirely sure how to address that yet”. Despite these concerns, most participants highlighted that birthday-banding remains a flexible and effective tool for managing competition structures and performance outcomes in a way that promotes effective player development. By recognising and addressing potential imbalances, stakeholders can refine the system to ensure that all players have access to appropriately challenging competitions and development opportunities.

### Building appropriate settings

Participants highlighted the critical role birthday-banding plays in building appropriate settings for players. More specifically, participants highlighted the capacity to promote flexibility and fluidity in grouping players because of the birthday-banding approach for both training and competition settings. Further, participants expressed the influential role birthday-banding plays on creating environments that encourage long-term player development.

#### Promoting flexibility and fluidity in groups

Generally, participants believed the birthday-banding approach provided flexibility in grouping players for training and competitions, while also enabling fluid movements for players across birthday-bands (i.e., playing-up or playing down) within the pathway. Participant 1 noted, “I try to avoid restricting players to just one group based on their age. Obviously, there are limits—you wouldn't pit a 17-year-old against a 10-year-old—but overall, I like to mix ability levels, and birthday-banding allows for that flexibility”. Alongside the flexibility to mix different levels of abilities and players together in the building appropriate training and competition environments, participants also highlighted the associated fluidity in moving players up and down the pathway. Participant 10 highlighted: “I believe in the importance of getting younger players to train with more experienced athletes. This approach [birthday-banding] promotes development, as long as everyone benefits and is challenged appropriately. It allows for fluidity in moving up and down the pathway, and I think younger players can gain a lot from training with their more experienced peers.” In comparison, Participant 9 felt that the traditional year-group system approach would create rigid divisions, where some players dominate while others fall behind, ultimately limiting the opportunities for appropriately building challenging environments to facilitate player development. Indeed, participants agreed with the flexibility and fluidity the birthday-banding approach allows in building training and competition environments to maximise developmental benefits for their players. Players can be moved up or down based on their development at the time, allowing them to face a diverse range of opponents (e.g., faster, stronger, smaller). This exposure challenges them to think strategically, likely enhancing their skill development.

Interestingly, participants also discussed the importance of the birthday-banding approach in building and maintaining competitive environments to ensure players are always appropriately challenged. Relating to the flexibility and fluidity to move players up the pathway, participants often noted how when these younger players moved into older pathways or groups, it created a competitive atmosphere pushing players to compete at a higher level. Further, in the context of competitive high-performance programmes, Participant 12 highlighted, “This flexibility [with birthday-banding] allows coaches to reassess players halfway through a season and bring them into the program if they show promise. It keeps everyone in the group on their toes, knowing that their positions aren't guaranteed and that there are players ready to step in”. Overall, participants felt that the birthday-banding approach, and its flexibility and fluidity to move players up and across different pathways, facilitated the building and maintenance of competitive environments to ensure players are always appropriately challenged to maximise player development.

Another advantage of the birthday-banding approach highlighted by participants was the fluidity of transition periods, both within and between groups. Birthday-bands span over a two-year period, whereby players generally remain in their respective band for that duration (e.g., a 12-year-old has two years before moving from an U13 birthday-band to an U15 birthday-band). This process over the 2 years for a player within a birthday-band was perceived by participants to help create a fluid transition into more challenging levels. Participant 15 highlighted, “I think the intention behind birthday-banding is to create a fluid system that facilitates learning and development. The first year serves as a transition year, allowing players to adapt as they move up age groups, where the game becomes faster, and rallies become longer. By grouping players within their brackets, we can ensure they are always competing against similar-aged peers”. Participant 15 also added, “Players essentially get two chances: one in their first year and a second opportunity in the next. This reflects the developmental expectations associated with performance”. Generally, participants believed that the birthday-banding approach created an environment encouraging fluid progression for players within their groups, while also providing opportunities for players to play across birthday-bands (i.e., playing-up and playing-down) if needed to maintain competitive environments.

Interestingly, participants also noted that many are leveraging the flexibility and fluidity of the birthday-banding approach to group players based on factors beyond age, to build more appropriate environments for player development. Participants 5 added, “In our coaching pathway, banding becomes more fluid and focuses more on skill, ability, and effort rather than age alone”. Participant 5 further highlighted how some regional competitions are based purely on ability and not age, stating “Teams are formed based on skill levels rather than age, merging ability-based methods with birthday banding where possible”. In fact, many participants referred to the birthday-banding approach as an “ability-based” approach during interviews, thereby underscoring this emphasis beyond age.

Given the fluid and flexible nature in grouping players with the birthday-banding approach, participants highlighted how this simplifies its implementation. Participant 10 highlighted, “Because birthday-banding is very open and fluid, it makes it quite simple to implement. It becomes more difficult if the governing body isn't fully behind it. Since this is a governing body decision, everything else can align and work accordingly”. In cases where there may be many players fitting into a certain category, or birthday-band, Participant 10 further suggested that organisations could consider breaking down groups into further ability-based categories within the birthday-band (e.g., elite and development level) to accommodate larger numbers. In an example from their context, Participant 10 noted, “For example, currently, we have two performance sections—development and potential—but also multiple age categories. Adjusting how organisations align their pathways with age categories could improve implementation”.

#### Creating an environment that fosters long-term development

Participants believed that the birthday-banding approach played a significant role in facilitating the creation of training and competition environments that foster long-term player development. Specific features of the training environment that were likely conducive to long-term player development was consistently highlighted by participants. For example, Participant 12 highlighted,

“I think it [birthday-banding] was to allow a more detailed look at individual players and focus on developing future talent. It provides young players with potential the opportunity to train with older, more successful athletes, which boosts their confidence. The group sizes are relatively small, enabling us to work closely with two or three players instead of cramming four or five into a session. This approach improves the quality of training and allows for specific skill development linked to the higher pathways, including the national junior and adult programs. Overall, it's been a beneficial strategy for individual athletes’ development”.

Participants generally believed that the environment created, empowered by the birthday-banding approach, allows for a more detailed focus on the individual encouraging long-term player development.

Another advantage highlighted by participants was that the birthday-banding approach encourages a shift away from age-based grouping, and towards building ability-based environments to encourage long-term player development. This connection was reinforced throughout the interviews, as many participants frequently referred to the birthday-banding approach as “ability-based” systems. Participant 10 highlighted,

“It [birthday-banding] allowed us to work with players operating at a younger age group who might not have made the squad if we were strictly using age categories. It gave us the chance to continue developing those players. We could also mix sessions based on ability rather than limiting them by age. This means a high-quality younger player could train alongside an older player if their abilities matched, removing those barriers”.

Participants also added that this approach allows players who may not be excelling now to have more time for development and to reach their potential. Further, by placing them in training environments that match their current ability, participants believed this created an environment which allowed players to still be part of the team and be given meaningful opportunities. This approach aligns with long-term development through building appropriate environments for the individual player.

When discussing the benefits of the birthday-banding approach, participants consistently highlighted the improved player retention rates. Many participants observed that more players remained involved in the sport for longer. Participants attributed the high player retention rates to the environment created, which was influenced by the birthday-banding approach. When discussing the different indicators for successful use of the birthday-banding approach, Participant 14 noted, “Retention and participation are the two main indicators. Once you hook them in, they tend to stay. More kids are showing interest in big tournaments, which leads to bigger draws and better facilities, giving everyone an opportunity to compete”. Relatedly, participants particularly continued to highlight the competitive environment available at every level for players as a possible explanation to the higher retention rates of players. Indeed, every player, whether excelling or still developing, has appropriate competition and opportunity to find a suitable challenge due to flexibility and fluidity of the birthday-banding approach. This fosters a sense of continuous challenge and progression pathways, encouraging players to stay in the sport long-term to fulfil and realise their potential. In relation to this, Participant 3 added, “You also see more players making it into the final stages of tournaments. The pyramid at the top is less narrow, and that gives a bigger base of players to choose from. More players can reach national or international levels”.

Expanding on player retention and participation rates, participants highlighted the effect of the birthday-banding approach on countering RAEs. Players born across different birth quarters are given equal opportunities because of the competitive environments created, specifically with tournaments spread out throughout the season. Participant 3 added, “It [birthday-banding] definitely seems to be working well in terms of relative age effects. There's a more even spread of participation across different birth quarters, whereas in other sports, you usually see a bias toward players born earlier in the year”. As a result, participants consistently highlighted birthday-banding as a fair approach for all players, regardless of RAEs, fostering an environment that supports long-term development.

In further highlighting the “fairness” associated with birthday-banding, participants believed the approach was the most appropriate way to allow players to progress and continue their journey in competitive sports. Participant 4 noted,

“It's meant to make things fair. You have to play in the age categories—under 11, 13, 15, and 17. You play a certain amount of time in the first season and then a full season in your last year, where you're probably at your strongest. For instance, my daughter just turned 13 in May, so now she's moved up into the next age category. She has two seasons to really improve her squash and achieve her goals”.

Participants added that training environments may already expose players to older opponents if appropriate, emphasising the fluid progression and the creation of environments tailored to support the long-term player development of the players. Importantly, Participant 3 highlighted the potential concern about differing systems across other sports that may be raised by parents (e.g., strict cut-off dates in other sports). However, Participant 3 added such concerns can be addressed through communication with key interest-holders of the players (e.g., parents) on the long-term benefits of birthday-banding for player development.

### Facilitating individual athlete development

Participants perceived the birthday-banding approach as a valuable system to foster individual athlete development, particularly in promoting holistic growth and progression. Participants also emphasised the importance of evaluating players' physical and skill development within the birthday banding approach to ensure continued development.

#### Encouraging holistic development and progression

Participants highlighted birthday-banding as a particularly important approach in facilitating the holistic development of athletes as they progress through the sporting pathway, including increased motivation, confidence, and resilience. These advantages were attributed to exposure to diverse training and competitive environments underpinned by the birthday-banding approach. Participant 13 highlighted, “Players become comfortable in their zones but also push their limits [with birthday-banding]. I see notable improvements when players mix with different standards. They gain confidence from competing against a range of abilities, which is essential for their growth”. Participants added that the birthday-banding approach allows for a more individual focus on players, enabling the monitoring and tracking of development more effectively. Participants 10 and 12 highlighted that the emphasis on results at a young age is not essential. Instead, the birthday-banding approach supports a medium- to long-term focus on individual player development, allowing athletes to progress into competitive pathways at older ages. In contrast, participants felt that the traditional fixed age-grouping system fosters a results-driven mentality within younger ages thereby not maximising holistic development and focusing on short-term results.

Another advantage of the birthday-banding approach noted by participants 6, 10, 12, and 13 is the allocation of more time spent between the coaches and players during their developmental stages. Given birthday-bands span for a 2-year period, coaches are likely to have a greater impact on the athlete. Participant 10 highlighted,

“It [birthday-banding] has helped us get to know the players better and spend more time with them. Players are part of the pathway more consistently, which allows for greater opportunities for development and impact. We can really help shape their careers and development in a consistent manner, which is a significant benefit”.

Participants also added that moving through different birthday bands exposes players to a variety of coaches, creating opportunities to maximise their talent and realise their potential. This diverse coaching experience is likely to help players develop a wide range of skills and abilities, ultimately supporting holistic development.

While most participants highlighted the advantages of holistic development underpinned by the birthday-banding approach, a few noted challenges players may face as they progress into higher birthday-bands and counter increased competition levels. Participants 3 and 9 discussed the challenges players may face when progressing into higher birthday-bands, particularly the mental impact. Indeed, as players enter a new birthday-band, they may no longer be competing at the high level they were accustomed to in their previous age group and must work their way back to the top. Participant 9 highlighted,

“There's also the confidence issue when players move through the ranks based on their birthdays, not necessarily because they're physically ready to do so. The really good players tend to make that transition more easily than those who aren't as strong. This can be off putting, and we may lose players who find that transition difficult and lose confidence”.

However, participants 9 and 3 added if such issues are managed correctly with the individual players, these barriers and issues can be addressed.

Relatedly, the progression of the individual athlete through the pathway is a crucial process that should be carefully evaluated to determine the best approach for their holistic development. Participant 2 highlighted, “It's about where the athlete will benefit the most in terms of their development. Sometimes it's better to challenge them by playing up, but other times staying in their age group makes more sense. It's a balancing act.” Ultimately, the birthday-banding approach offers flexibility in considering the aspect of player progression, allowing coaches to assess whether moving up is the in the best interest of the individual player's holistic development, something that the traditional age-grouping approach does not provide.

#### Evaluating physical and skill development

Participants highlighted that physical and skill development should be consistently evaluated and considered to help support the ongoing development of individual athletes within the birthday-banding approach. Players may dominate the sport early on due to physical advantages (e.g., size or strength), but these short-term benefits may not necessarily guarantee long-term success. As players mature other aspects of their game will likely become more critical (e.g., technique, skill, and mental factors), therefore by not addressing the physical advantages, the individual player's development may not be maximised. Participant 13 highlighted, “Birthday-banding avoids the issue of older, stronger players dominating due to physical maturity. It allows for a mix of players to come into the age group at different times. This diversity means that players are exposed to various standards and coaches, promoting development”. Indeed, most participants viewed the birthday-banding approach as highly beneficial in countering physical advantages for individual players.

However, one participant pointed out that significant differences in physical development still exist within the birthday-banding approach and can negatively impact individual player development. Participant 1 added,

“For instance, there could be a situation where someone is physically stronger and more developed, just because they were born earlier in the cycle. You might have someone who's very young and facing a two- or three-year difference, which creates a huge gap. This disparity might discourage some juniors from participating or trying to get involved. On the flip side, you might have juniors who are excelling in their age group but are bigger and stronger. As the age groups go up, they might get demoralised because they start losing to someone two or three years younger than them”.

This perspective highlights the importance of carefully managing physical development of the individual athletes through competitive and training environments. All participants strongly viewed the birthday-banding approach as highly beneficial in managing and supporting the physical and skill development of individual athletes to ensure continued development. Given the flexibility to move players across the pathway according to their development, or their advantages at the time, birthday-banding is a highly beneficial approach to help manage these disparities.

Importantly, Participant 15 noted, “It's important to look past immediate advantages and consider long-term prospects. We see juniors performing well in competitions at the under-13 level, but sometimes they fade out by the time they reach the under-17 level. For example, a player who is a big hitter might initially excel due to physical and technical advantages, but these weaknesses can become more apparent as they mature. As an organisation, we need to avoid focusing solely on the ‘big kid’ and instead assess each player's unique attributes”. Participants perceived the birthday-banding approach allows coaches and key interest-holders within the individual player development process to consider the individual player and focus on their long-term potential rather than short-term successes (e.g., a player with physical advantages). The flexibility of the birthday-banding approach allows players to be matched together according to their physical and skill development, ensuring appropriately challenging environments to optimise individual athlete development.

## Discussion

The current study explored the operational mechanisms of the birthday-banding approach in the England Squash Talent Pathway and potential associations with player development outcomes according to the perceptions of the coaches embedded within. The findings highlighted coaches believed birthday-banding, which has been previously shown to eliminate RAEs in this specific sports setting, has a significant impact on talent identification and development processes. To the authors' knowledge, this is the first study to qualitatively assess coaches' perspectives on this novel approach to address RAEs, and the results provide valuable insights into how birthday-banding may influence environmental, individual, and organisational components of athlete development.

The findings highlighted how birthday-banding influences organisational structures and environmental factors, particularly in terms of competition and selection processes. Participants indicated that the approach not only mitigated biases but also helped to create a more inclusive, flexible, and fluid competition framework. This flexible grouping allows coaches to tailor the athlete experience and selection processes based on a range of factors beyond age to ultimately ensure that players are given appropriate developmental opportunities, and are selected based on attributes the coaches believe are important for long-term development rather than being constrained by current performance metrics. This is particularly important, since previous research has highlighted that the primary challenges and pitfalls of talent identification and athlete development systems are generally centred around the emphasis on short-term, performance-related outcomes (50). By fostering these tailored, adaptive environments, birthday-banding may offer athletes a more equitable experience that promotes their long-term development.

At the individual level, coaches strongly emphasised how birthday-banding facilitates holistic athlete development. This suggests the approach not only enables physical development, but also addresses psychological, technical, and tactical aspects that are important and perhaps become more so at older ages and higher competitive playing levels. Indeed, many studies have emphasised the importance of considering multidimensional factors in talent identification and athlete development settings [e.g., (51)], including preliminary research in squash [e.g., (52)]. By reducing the pressure on relatively younger athletes who may otherwise be overlooked due to their age, coaches suggest birthday-banding creates a more balanced approach to player development that considers a wider spectrum of growth and maturation nuances. As squash players progress through the talent pathway, the holistic development fostered by birthday-banding may help to ensure that athletes have the opportunity to realise their potential, rather than being prematurely pigeonholed based on their early/late birthdate.

While the findings from this study are specific to squash, the implications of birthday-banding may extend to other sports. As demonstrated by the consistent presence of RAEs in youth talent development systems across many sports, including other racket sports (e.g., badminton, table tennis, tennis) (25), birthday-banding could represent an innovative solution to the biases introduced by fixed chronological age cut-off points. The absence of RAEs in squash suggests that sports which currently experience challenges related to RAEs might benefit from adopting a similar model, particularly those whose athletes experience significant developmental pressure at younger ages. Moreover, the flexibility inherent in birthday-banding could also be applied to coaching frameworks, where athletes are not categorised based solely on age but rather on their readiness to perform at various competitive levels (e.g., playing-up and playing-down). By reducing biases related to RAEs, birthday-banding could create more fluid pathways to development, especially in sports where early maturation often provides an advantage. In addition to birthday-banding, it is also important to acknowledge other possible approaches to group athletes. For example, bio-banding can be used to reduce maturation-based inequalities by grouping athletes according to biological markers or distributing opportunities more equitably across the year (53, 54). Research has shown that stakeholders perceive these models as beneficial for talent development and psychological engagement (55). While the implementation differs from birthday-banding, the underlying rationale is similar, by ensuring fairness and developmentally appropriate environments throughout youth sport pathways (56).

How the coaches viewed birthday-banding, through its operational mechanisms and the associated player development outcomes (i.e., environmental, individual, organisational) aligns with Kelly et al.'s (15, 51) proposal that the Personal Assets Framework (PAF) is a useful model with which to explain the potential mechanisms of RAEs on youth development. According to research in developmental and sport psychology, the PAF identifies three essential “dynamic elements” necessary for sport development to take, including: (a) personal engagement in activities (i.e., the what), (b) appropriate settings and organisational structures (i.e., the where), and (c) quality social dynamics (i.e., the who) (57, 58). The interactions of these elements create an immediate sporting experience that can impact developmental outcomes in the short-term, such as character, confidence, connection, and competence (i.e., the 4Cs), as well as participation, performance, and personal development (the 3Ps) in the long-term. The PAF, therefore, could perhaps be used as a heuristic to help frame discussions regarding the mechanisms of RAEs as well as attempted solutions (e.g., birthday-banding) at mitigating this (un)conscious bias.

## Limitations and future directions

Although the current study provides important insights into the potential mechanisms that underpin birthday-banding, it is not without limitations. First, the sample size of 15 coaches, while offering valuable perspectives and a large proportion of coaches in the England Squash Talent Pathway, may not fully represent the diversity of coaching experiences. Future research could expand this sample to include more coaches, female coaches, and other interest-holder such as the athletes themselves to provide a more comprehensive understanding of the approach's impact. Additionally, while the qualitative nature of this study allowed for an in-depth exploration of coaches' perceptions, it would be valuable to investigate the tangible outcomes of birthday-banding in terms of performance metrics, injury rates, and long-term player retention. Quantitative data could offer more empirical evidence to support the efficacy of this approach. Future studies could also compare the outcomes of players within the birthday-banding system against those in traditional age-based groupings to more rigorously evaluate the impact on player development. Importantly, some coaches raised concerns with the ways in which birthday-banding can go wrong. These concerns were compiled as a way to provide a critical evaluation of the approach, and suggestions have been made on ways to minimise their impact on the system, to further improve the birthday-banding approach.

Although our findings suggest birthday-banding could be beneficial for squash in England, its transferability may vary significantly depending on the sport and country. In disciplines with high early-specialisation, dense competition calendars, or where ranking-based selection is predominant (e.g., football, gymnastics, swimming), the effectiveness and feasibility of birthday-banding may be constrained. For instance, Pérez-González et al. (59) have demonstrated varying relative age magnitudes across European women's football leagues, highlighting how institutional structures and competitive density influence relative age dynamics. This suggests that birthday-banding should be applied with caution and adapted to sport-specific environmental and organisational contexts.

## Conclusion

Generally, sport policy makers are largely responsible for RAEs, due to the group banding policies that are often implemented within youth sport. As decision-makers, and stakeholders in youth sport, we have the capability and responsibility to look beyond fixed age group structures to try and create more developmentally appropriate settings for every young person to achieve their potential. With this in mind, coaches from England Squash offered a unique insight into their birthday-banding approach, showing the possible environmental, individual, organisational benefits. This study also contributes to the growing body of literature on RAEs and provides novel insights into how birthday-banding can help mitigate these biases in the England Squash Talent Pathway. By shifting away from traditional age-based groupings and focusing more on developmental milestones, birthday-banding appears to foster a more equitable, flexible, and holistic approach to athlete development. The coaches in this sample perceived the birthday-banding approach to be an easy-to-implement way of creating an ability-focused environment that affords more opportunities to athletes to experience challenge and success throughout the developmental pathway. While further research is needed to confirm the long-term effectiveness of this approach, the current findings suggest that birthday-banding may be a valuable solution, not only for squash, but also for other sports seeking to address RAEs and optimise their talent development systems.

## Data Availability

The original contributions presented in the study are included in the article/Supplementary Material, further inquiries can be directed to the corresponding author.
